# Authors and editors assort on gender and geography in high-rank ecological publications

**DOI:** 10.1371/journal.pone.0192481

**Published:** 2018-02-08

**Authors:** Kezia R. Manlove, Rebecca M. Belou

**Affiliations:** 1 Washington State University College of Veterinary Medicine, Pullman, WA, United States of America; 2 Montana State University Office of Planning and Analysis, Bozeman, MT, United States of America; Institut Català de Paleoecologia Humana i Evolució Social (IPHES), SPAIN

## Abstract

Peer-reviewed publication volume and caliber are widely-recognized proxies for academic merit, and a strong publication record is essential for academic success and advancement. However, recent work suggests that publication productivity for particular author groups may also be determined in part by implicit biases lurking in the publication pipeline. Here, we explore patterns of gender, geography, and institutional rank among authors, editorial board members, and handling editors in high-impact ecological publications during 2015 and 2016. A higher proportion of lead authors had female first names (33.9%) than editorial board members (28.9%), and the proportion of female first names among handling editors was even lower (21.1%). Female editors disproportionately edited publications with female lead authors (40.3% of publications with female lead authors were handled by female editors, though female editors handled only 34.4% of all studied publications). Additionally, ecological authors and editors were overwhelmingly from countries in the G8, and high-ranking academic institutions accounted for a large portion of both the published work, and its editorship. Editors and lead authors with female names were typically affiliated with higher-ranking institutions than their male peers. This description of author and editor features provides a baseline for benchmarking future trends in the ecological publishing culture.

## Introduction

Mounting evidence shows that implicit biases structure academic performance metrics [1–4] and institutional hiring practices [5]. These biases may also operate along the peer-review pipeline to inadvertently constrain the diversity of perspectives represented in the peer-reviewed literature. The gate-keeping role of academic journal editors makes them uniquely positioned to advance minority perspectives. Editors and editorial board members are likely subject to the same implicit biases that work against women and geographic minorities in other settings [6]. However, the role that implicit bias could play in the publication pipeline has not been extensively explored in the ecological literature.

Diverse representation across genders and geographies is particularly critical for ecology and evolution, since research questions in these domains regularly stem from context- and individual-specific observations [7–9], and inferences may vary dramatically among research locales. Despite the importance of diverse perspectives in ecology, minority groups have historically been underrepresented among editorial boards in environmental and conservation journals [10]. Moreover, multiple-minority groups (for example, women working at institutions outside the economic Group of Eight (“G8”) are disproportionately underrepresented in scholarly communications in general [11]. There is limited empirical synthesis of gender or geographic patterns in the ecological publication culture (but see [12–14]. In particular, little work addresses questions about how editor attributes—apart from area of research interest—relate to those of authors (but see [15,16] for a summary from *Functional Ecology*).

Here, we analyze patterns of gender, geographic locale, and institutional rank among editorial board members, handling editors, and lead authors publishing in high-impact ecological journals. In addition to describing author and editor attributes, we also test the following hypotheses. First, that women and geographic minorities are better represented among lead authors than among editorial board members, and better represented among editorial board members than among handling editors of published manuscripts. Second, that editors assort with lead authors by gender, geography, and institutional rank, with female editors disproportionately handling publications led by women; editors from developing economies disproportionately handling contributions led by authors from these same economies; and editors from high-rank institutions disproportionately handling work led by authors from high-rank institutions. Finally, that women who are active throughout the publication process come from higher-ranking institutions than their male peers.

## Methods

### Researcher populations and attributes

We characterized and compared three distinct researcher populations: lead authors, editorial board members, and editors credited with handling published papers (“handling editors”). For each population, we considered three attributes: gender, affiliation with an institution in a G8 country, and rank of the affiliated institution. Sampling methods for each population are described below. Throughout the manuscript, we use “minority” to reference any group with attributes distinct from those of the most common group (generally, researchers with traditionally male first names from institutions located in G8 countries, though this group was only a plurality and not a majority in some cases).

### Data collection

We identified a set of high-impact ecology journals in November, 2016 using the SciMago journal rankings (http://www.scimagojr.com/journalrank.php) with subject area “Ecology, Evolution, Behavior, and Systematics”. We excluded journals that limited their publications to invited manuscripts (journals like *Annual Review in Ecology*, *Evolution and Systematics*, *Annual Review of Entomology*), and journals that did not explicitly credit the handling editor on published manuscript (journals like *Trends in Ecology and Evolution*, *Genome Biology*, *Frontiers in Ecology and the Environment*, *Global Change Biology*, *The ISME Journal*, *BMC Biology*, *Molecular Ecology*, *Fish and Fisheries*). We further limited our analysis to 10 high-impact journals that represented a diversity of ecological interest areas, produced through a variety of publishers. Study journals are listed in Table 1.

**Table 1:** Summary of queried journals.

Journal Name	Publisher	Number of Papers included (total papers published during sampling window)	Journal Impact Factor (2016)*
Ecological Monographs	Ecological Society of America (Wiley)	61 (61)	8.759
Ecology	Ecological Society of America (Wiley)	335 (628)	4.809
Ecology Letters	Centre National de la Recherche Scientifique (Wiley)	282 (282)	9.449
Global Ecology and Biogeography	(Wiley)	271 (271)	6.045
Journal of Animal Ecology	British Ecological Society (Wiley)	276 (327)	4.474
Journal of Ecology	British Ecological Society (Wiley)	323 (323)	5.813
Methods in Ecology and Evolution	British Ecological Society (Wiley)	274 (322)	5.708
Molecular Biology and Evolution	Society for Molecular Biology and Evolution (Oxford Academic)	300 (569)	6.202
PLoS Genetics	Public Library of Science	240 (1,422)	6.100
Systematic Biology	Society of Systematic Biologists (Oxford Academic)	160 (160)	8.917

*Journal impact factors obtained from the InCites module associated with Web of Knowledge.

We used Web of Science to extract all papers published in our study journals during 2015 and 2016 through a set of searches run in late November and early December, 2016 (these searches usually captured papers up through each publication’s November 2016 issue). When searches returned less than 300 manuscripts, all manuscripts were included in the dataset. When searches returned more than 300 manuscripts, we systematically sampled the returned papers chronologically, in blocks of 50, so that each journal contributed approximately 250–350 papers (with the notable exception of *Ecological Monographs* and *Systematic Biology*, which published a lower volume of work than the other study publications; Table 1). Editorial board membership was extracted from journal websites during this same time period.

We returned complete bibliographic information for each publication from Web of Science, including author names and institutional affiliations. Since Web of Science does not include editor information, we accessed pdfs of all sampled papers to extract the editor’s full name. We parsed the Web of Science author institution field to return a character string describing the lead author’s institutional affiliation (e. g., “University College London”), and extracted editor institutional affiliation information from the journal editorial board websites. We used Wikipedia’s GeoHack tool to identify the city where each institution was located. Wherever possible, we extracted coordinates from Wikipedia for the institution itself (in latitude/longitude), but on occasion, we were limited to coordinates of the institution’s city. We then classified institutions according to whether they were located within countries in the G8 economic group (Canada, France, Germany, Italy, Japan, Russia, the United Kingdom, or the United States). We excluded 199 papers from the geographic and institution rank analyses for which lead author or handling editor institutions could not be uniquely identified, or placed geographically.

We ran all lead author and editor first names through a gender classifier implemented in the statistical computing environment R (package gender [17], function gender, using method = “kantrowitz”). We cross-checked that classification method’s results with classifications based on a US social security database (package gender, function gender, using method = “ssa”). We reviewed all names that gender left unclassified, along with all cases where the two classifiers disagreed, by hand, and used a variety of baby naming websites to obtain a gender classification for an additional 15% of names.

We used the 2016 Shanghai rankings system (http://www.shanghairanking.com/ARWU2016.html) to identify the top 200 global institutions in the life sciences. We extracted a categorical institution ranking (in the top 200 or not) for all authors’ and editors’ institutions. We also built a numeric rank variable, which was integer-valued for institutions ranked among the top 50; group medians for institutions ranked between 51 and 200 (since the Shanghai ranking only provides ranking blocks at that level, e.g., 51–75, 76–100, 101–150, 151–200); or fixed to “250” for un-ranked institutions outside the top 200. For example, all institutions ranked in the 51–75 block were assigned a rank value of 63. We used this numeric rank to build two additional indicator variables for institutions ranked in the top 50 and top 100.

### Analysis

#### Comparing author, editorial board, and handling editor attributes

We compared the handling editor, editorial board, and lead author populations in terms of their gender composition, geographic structure, and institutional ranking. We used a logistic regression model with hierarchical effects (S1 Supporting text) to compare rates of female participation as editorial board members, handling editors, and lead authors, in a model also adjusting for journal-to-journal variation with a random intercept. To test whether institution rank differed among handling editors, editorial board members, and lead authors, we used a generalized linear mixed effects model with a quasi-Poisson residual structure and random intercepts for journals, implemented through MASS’s glmmPQL function. We used a hierarchical logistic regression model identical in structure to the one used for researcher gender to compare researcher roles (i.e., lead author, handling editor, editorial board member) for individuals from G8 and non-G8 countries.

#### Assessing editor-author assortativity

We first examined assortativity from the perspective of the editor, asking whether female editors disproportionately handled papers by female lead authors. We used a Chi-square test to determine whether handling editor gender varied significantly with gender of the lead author, and examined deviations from Chi-square expectations to identify over- and under-represented combinations of author-editor features.

Since some researchers’ genders could not be assigned with high precision, we ran this analysis twice, first ascribing individuals with unassigned gender to a separate “either” category, and then limiting the analysis to only those publications with high-precision gender assignments for both the lead author and the editor. We constructed bootstrapped confidence intervals on the proportion of editor/author assortativity by resampling papers within each editor group (papers edited by editors with unclassified genders, female editors, and male editors), cross-tabulating lead author gender with editor gender for each resampled dataset, and refitting the Chi-square test to the resampled data. In each resampling, we extracted the Chi-square residual value (“observed” value—“expected” value). We then replicated the entire analysis from the perspective of the lead author, asking whether authors with particular attributes were more likely to have their manuscripts handled by editors from those same groups.

We used an identical approach to compare institutional ranks and affiliation with institutions in G8 countries for lead authors and their handling editors.

#### Relationship between minority contributions and institution rank

We compared the proportion of researchers from top-100 institutions by researcher gender for each of our focal populations (lead authors, editorial board members, and handling editors) by fitting three generalized linear mixed effects models, one for each focal population. The models considered institutional rank as a function of individual’s gender, with a random intercept adjusting for journal-to-journal variation.

All analyses were conducted in the R statistical computing environment [18]. The complete dataset and corresponding code are provided in S1–S4 Data files. In all cases, individuals were allowed to enter the analysis multiple times if they contributed to the database more than once (for example, if an individual were lead author or handling editor on multiple papers, or if an individual led a paper and also served on an editorial board).

## Results

### Data description

Our publication bank included 2,532 papers. 2,447 of those papers listed editors and 2,430 included the lead author’s full first name (the others included only lead author first initials), leaving us with 2,351 papers with both author and editor first names. We also acquired data on all 904 editorial board members listed on each journal’s webpage.

The distribution of gender, institutional rank, and country’s presence in the G8 for each population—lead authors, editorial board members, and handling editors—are shown in Figure A in S1 Supporting text. Automated gender classifiers and follow-up searches allowed us to assign a likely gender to 87% of lead authors, 95% of handling editors, and 95% of editorial board members (proportion of automated and manual classifications for each group are described in S1 Supporting text). In total, we developed gender classifications for both lead author and editor on 1,964 of the 2,532 study papers. For simplicity, we refer to model-assigned gender as “gender” throughout the results. For example, the phrase “female handling editors” should be interpreted as meaning “handling editors who were identified as having female first names under our gender identification procedures”.

For 2,515 of the 2,532 papers, lead authors institutional affiliations could be identified to particular countries. Of these, 58% came from G8 nations. For handling editors, this proportion rose to 73%, and for editorial board members, it increased further to 74%. 92% (184) of the 200 highest-ranking life sciences institutions were represented by at least one individual across the three study populations (lead authors, editorial board members, or handling editors). Top-200 institutions housed 45% of lead authors, 49% of editorial board members, and 50% of handling editors in the dataset (Table 2). While 25% (626 / 2,515) of lead authors came from institutions outside North America and Europe, this was true for only 17% of editorial board members (150 / 904) and 18% of handling editors (450 / 2,510).

**Table 2:** Summary of sampled data.

Population	Total (Unique)	% Female* (number classified as M/F/ Either)	% from top-200 institutions	% from G8
Lead authors	2430 (2426)	33.9–44.7% (1134/695/223)	47.9% (1125 of 2349)	61.8% (1452 of 2349)
Editorial board members	904 (867)	28.9–37.0% (542/249/69)	48.6% (436 of 898)	73.9% (664 out of 898)
Handling editors	2447 (743)	21.1–29.4% (1638/490/193)	52.1% (1251 of 2401)	75.9% (1821 out of 2401)

For % female, the lower number includes only individuals with names classified as “female”, while the higher number includes both “female” and “either” classifications in the numerator. Denominator numbers vary slightly across rows, since some individual names, institutions, or continents could not be classified. Accordingly, we report denominator values in parentheses throughout.

*Lower value reflects estimate if all “either” classifications are treated as male. Upper bound reflects estimate if all “either” classifications are treated as female.

54.6% (494 / 904) of individuals listed on editorial boards were credited with handling at least one published paper, while 30.8% percent (779 / 2532) of individuals who credited with handling publications were not listed on editorial board websites at the time of data collection.

The distribution of number of papers handled per editor was highly overdispersed, regardless of handling editor gender (Figure B in S1 Supporting text), with the most productive 7% of editors handling 24% of publications, and the least productive 70% of editors handling less than 40% of publications. Global maps of author and editor activity levels are shown in Figure C in S1 Supporting text.

**Fig 1:**
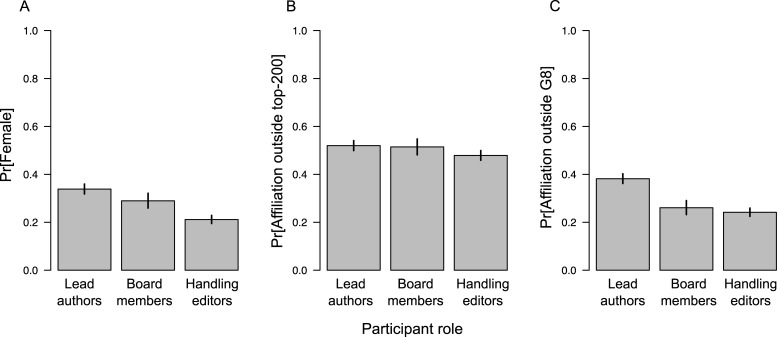
Data structure. Gender, institutional rank, and G8-status for lead authors, editorial board members, and handling editors across studied publications. Bars extend to 95% binomial confidence limits for all estimates.

Attribute summaries for lead authors, editorial board members, and published editors are shown in Table 2 and Fig 1.

### Researchers with minority attributes were less-represented among handling editors than among editorial board members or lead authors

**Fig 2:**
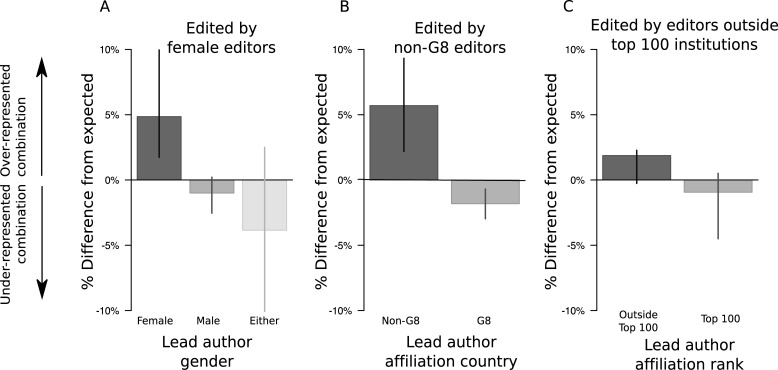
Assortativity patterns between handling editors and authors. Error bars extend to 95% bootstrapped confidence intervals. The author group mirroring the editor group on each attribute is always furthest to the left. In all cases, handling editors disproportionately handle papers by lead authors with similar traits. The 0% line is the expected value under the Chi-squared test.

Researchers with female names made up a larger proportion of lead authors than editorial board members or editors of published manuscripts (33.9%, 28.9%, and 21.1%, respectively; Table 2, Fig 2A). The logistic regression of female participation as a function of role (author, board member, handling editor) showed women acted as lead authors at significantly higher rates than in either of the editorial roles, after accounting for differences among journals ($\hat{\beta }$ = -0.193, p = 0.025 when comparing the proportion female board members to a lead author baseline group; $\hat{\beta }$ = -0.499, p <0.001 when comparing the proportion of female handling editors to a lead author baseline group; Table B in S1 Supporting text). The quasi-Poisson model suggested institutional rank for lead authors did not differ significantly from that of editorial board members ($\hat{\beta }$ = -1.940, SE = 4.164, p = 0.600; Table C in S1 Supporting Text), though handling editors came from significantly higher-ranked institutions than lead authors ($\hat{\beta }$ = -8.168, SE = 3.700, p = 0.003). Lead authors were much more likely to be affiliated with institutions outside the G8 countries than were editorial board members or handling editors (Fig 1; Table D in S1 Supporting text; $\hat{\beta }$ comparing proportion of board members from non-G8 country to proportion of lead authors from non-G8 countries = -0.322, SE = 0.084, p < 0.001; $\hat{\beta }$ comparing proportion of handling editors from non-G8 countries to proportion of lead authors from non-G8 countries = -0.485, SE = 0.061, p < 0.001).

### Editors and authors assorted strongly by gender and geography, and weakly by institutional rank

We examined gender assortatitivity patterns on the 1963 papers for which lead author and editor gender were both classified. The distribution of lead author genders on handled papers varied marginally (X^2^ = 8.555 on 4 df, p = 0.073) among handling editor gender groups when an “either” gender classification was included, and this effect became significant (X^2^ = 5.949 on 1 df, p = 0.015) when papers with “either" gender classifications were removed.

Overall, 34.4% of the 1963 publications in the gender assortativity analysis had female lead authors, but among publications handled by female editors, this rose to 40.3% (Fig 2A). Overall, 20.5% of publications had female handling editors, but publications by female lead authors had female handling editors 24.0% of the time, whereas publications by male lead authors had female handling editors 18.7% of the time.

Editors and authors also assorted on national economic rank (X^2^ = 10.812 on 1 df, p = 0.001; Fig 2B). Overall, 75.8% of publications were edited by G8 editors, but that number fell to 70.2% among publications led by non-G8 authors. Editors and authors did not assort significantly on institutional rank (X^2^ = 1.959 on 1 df, p = 0.162; Fig 2C). Continental assortativity was evident, and this was particularly true of editors and authors from Asian institutions (Figure D in S1 Supporting text).

### Researchers with female names were affiliated with higher-rank institutions than researchers with male names

**Fig 3:**
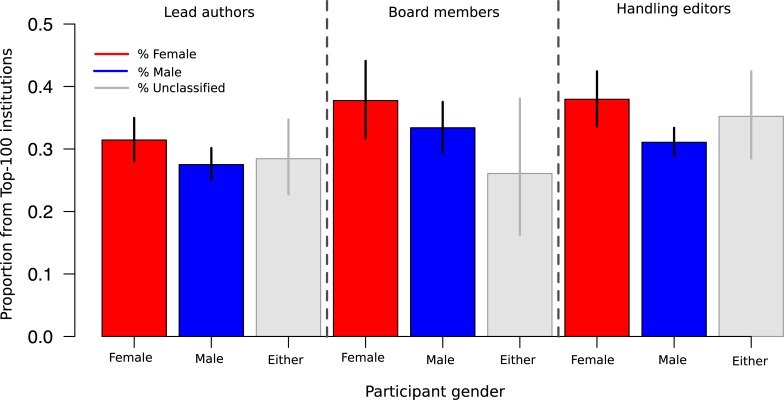
Gender by institutional rank. Proportion of participants from each population that represent highly-ranked institution, according to gender classification.

Female lead authors and handling editors both came from significantly higher-ranked institutions than their male counterparts, after accounting for country- and journal-specific effects, and this was also marginally true for board members (Fig 3). The odds that a female lead author came from an institution ranked in the top 100 was 1.25 times the odds that a male lead author came from a top-100 institution (95% CI [0.999, 1.559]; Table E in S1 Supporting text). Among handling editors, the odds a female lead author came from a top-100 institution was 1.57 times the odds a male lead came from a top-2100 institution (95% CI [1.228, 2.012]), again after adjusting for journal and country (Table F in S1 Supporting text).

## Discussion

Maintaining diverse research perspectives is important for ecology and evolution, domains where geographic context and individual experience could feasibly shape both research questions and measured responses. In order to enter the accepted ecological canon, diverse perspectives should be represented in high-impact publications. However, publication hinges not only on the work’s merit, but also on a peer review structure potentially subject to implicit biases that systematically undervalue research produced by certain author groups. We analyzed editor and lead author data from 10 highly respected ecological journals, and found consistent patterns between lead author and editor traits, including gender, institutional ranking, and national economic status.

While many of the editorial boards from journals in this study report advances in recruiting women and minorities into their ranks over recent years [16, 19], we nonetheless find that women and minorities were still significantly more likely to author published papers than to edit them (Fig 1). Furthermore, minority editorial board members were less-likely to serve as handling editor on publications than their majority peers (Fig 1). Editor and lead author attributes showed significant (or nearly significant) assortativity in terms of gender, country’s G8 status, and institutional rank (Fig 2). Finally, we saw evidence that female lead authors and editors were more likely to be affiliated with high-rank institutions than their male peers (Fig 3).

**Fig 4:**
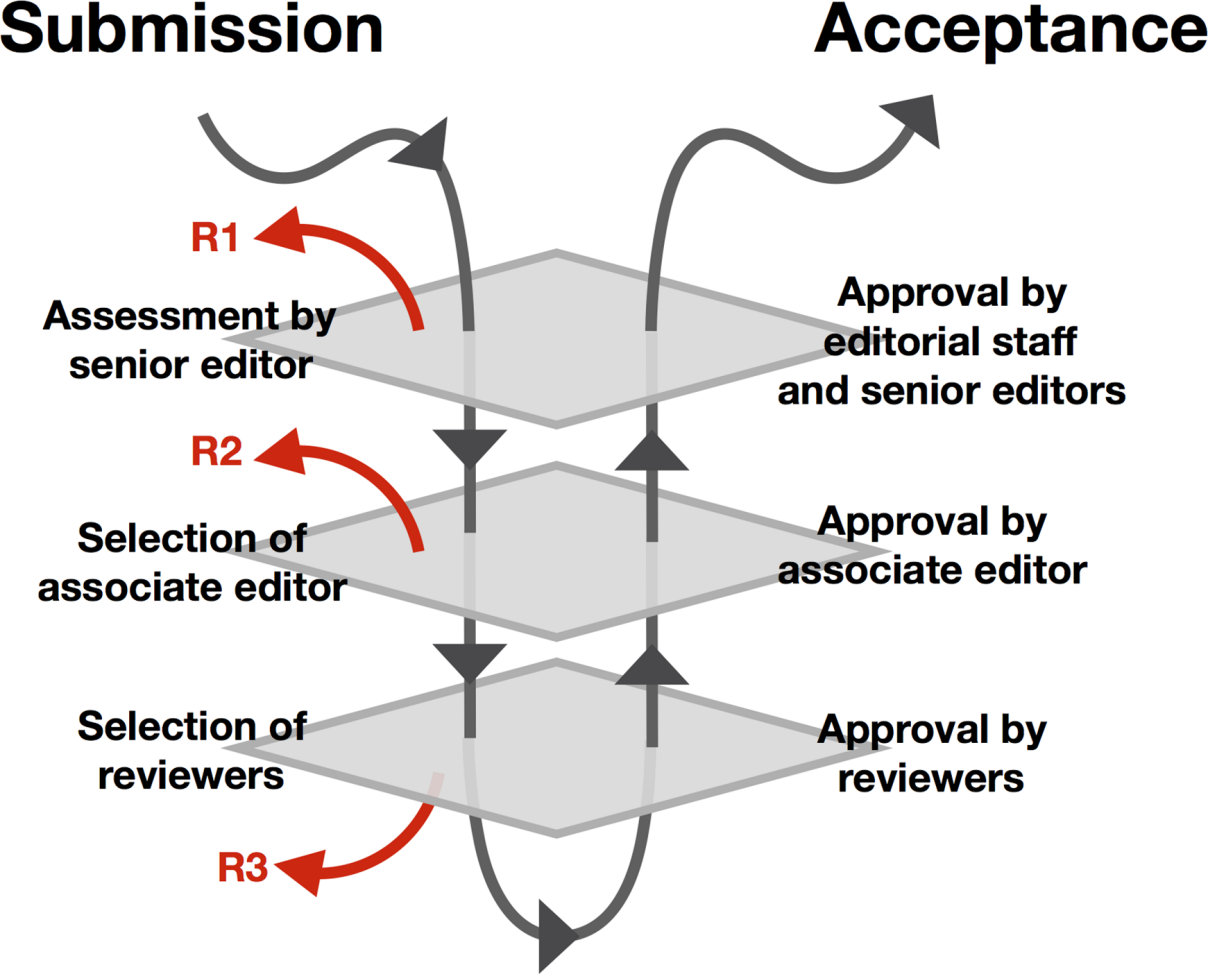
Potential stages of manuscript rejection in the peer-review process. R1 indicates rejections by senior editors prior to selection of an Associate (e.g., “handling”) editor. R2 indicates rejection by the Associate editor prior to circulation among peer reviewers. R3 indicates rejection by peer reviewers. A double-blind peer review process adjusts for biases operating at the R3 stage, but does not necessarily alter potential biases arising at the R1 or R2 stages.

Our findings point to places where editor characteristics could play a role in shaping peer review, a previously identified gap in the empirical literature on scientific publication [20]. Editors are known to have highly variable rejection rates [21], with female associate editors showing systematically higher overall rejection rates than their male peers in several subject areas and eras (see for example [22] for an analysis of editor rejection rates at *Journal of Neuroscience*, and [23] for a similar analysis of rejection rates at *Journal of the American Medical Association*). “Double-blind” peer review may mitigate implicit bias among reviewers (e.g., R3 in Fig 4) and possibly increase representation of female authors [12], but it is not generally clear whether blinding protocols are also applied to handling editors (e.g., R2 in Fig 4). Moreover, it is likely impossible to apply blinding to senior editors (R1), especially for manuscripts that rely on author credentials within the field as justification for consideration. In general, we did not find clear documentation of how handling editors were assigned among our study journals. Such transparency might be a first step toward mitigating any potential biases among editors, or alleviating unfounded author concerns thereof.

The assortativity patterns reported here could be due to minority groups “extending the ladder” [24, 25], either by authors disproportionately requesting editors with similar traits, or by editors disproportionately ascribing merit to work from authors they resemble. In the absence of data on handling editor rejection patterns, we cannot separate these mechanisms here. It is worth noting, however, that other analyses found women (and especially female postdoctoral researchers) to be particularly harsh reviewers [26]. It is plausible that the patterns reported here could arise from patterned editor rejections, as opposed to patterned editor assignments, and that question should be addressed through future study.

Gender is only one aspect of this exploration, and our geographic and institutional rank results may be even more important to ecological publishing. While understanding a particular ecological context likely facilitates article critique for many ecology manuscripts, there is evidence from domains where spatial context matters less that reviewers exhibit biases on the basis of author nationality. For example, US-based reviewers for the journal *Gastroenterology* systematically scored work by US authors higher than reviewers from outside the US [27]. If such country-level biases existed in the ecological literature, they could drive discrepancies in research volume across biomes (as is indeed already known to exist in the ecological literature [28]). In this analysis, only one of the 904 studied editorial board members represented an African institution, and that editor did not handle any paper in our dataset.

Taken together, our results provide an empirical argument that participant attributes could shape the peer review process at multiple levels in the high-impact ecological literature (Fig 4). Recent analyses of data from the American Geophysical Union showed that women were less-likely to be suggested as manuscript reviewers [29], and this trend may apply to handling editors and reviewers at ecological journals as well. Studies about the relationship between double-blind peer review and female participation rates have produced variable results [20], although [26] found that author gender made no difference in manuscript quality scores or reviewer decisions (though notably, their study focuses on reviewer, as opposed to editor, traits). Alternatives to double blind review, like entirely transparent “open review” processes, may alleviate some of the problems associated with single- and double-blind peer review, although it seems plausible that minority reviewers may accrue penalties for voicing criticism through review than their majority peers. Additional investigation into how these contrasting strategies drive structure in the review process is merited.

### Study limitations

A number of factors constrain the scope of inference to which our results apply. First, our sample was limited to only those journals that made editor and author names available in full. In practice, this meant that some publication families were over-represented (especially Ecological Society of America publications and British Ecological Society publications), and others (BMC, Cell) were excluded. It is possibly, and perhaps even likely, that publication practices are not independent of a journal’s decision to publish editor names, but that issue is beyond the scope of the present work.

Second, the gender classifier we used is optimized for North American and European names, and classification errors are likely higher for names originating in other cultures. While we did our best to circumvent this issue, it nonetheless limits our ability to separate geographic and gender effects. However, we are not certain as to how this effect might propagate in the publication culture. It is likely that individuals with names that elude the classifier are also less-likely to be classified (implicitly) into gender groups by editorial board members. The implication may be that individuals with unclassifiable names are not subject to the same implicit biases as individuals whose names classify with high probability to a particular gender group. However, this is pure speculation, and merits additional follow-up work.

Finally, this study is fundamentally cross-sectional. An investigation of temporal patterns in ecological publishing is beyond our current objectives, though it would undoubtedly shed additional nuance on our results. As it stands, this cross-section is intended to provide a published benchmark against which future patterns can be assessed.

### Conclusions

Regardless of the review tactic taken, the ecological publishing community might benefit from more transparency in the editor and reviewer assignment processes, accompanied by deliberate advancement of minority groups into roles of leadership [30]. Previous work has shown that sunshine is a powerful disinfectant for the sorts of biases that could underlie the patterns reported here [24]. We hope this assessment raises awareness about consistent yet latent patterns in the existing publication culture. Even background awareness about editor-author interactions may reduce resulting biases moving forward. The journals studied here have all made efforts in recent years to add diversity to their editorial staffs [19], and the ecological research culture continues to make great strides to mitigate biases. We hope that this analysis encourages authors to proactively consider recommending editors with gender and institutional features different from their own, especially in cases where those features have limited implications for the science at hand.

## Supporting information

S1 Supporting text Supplementary data descriptions, analysis, and figures. (DOCX)

S1 Data file Ecological metadata file for the papers, board members, and instititutions datafiles used in this manuscript’s analyses. (DOCX)

S2 Data file papers.csv file with records for all sampled papers included in this analysis. (CSV)

S3 Data file board members.csv file with records for all editorial board members included in this analysis. (CSV)

S4 Data file institutions.csv file with records for each institution included in this analysis. (CSV)

S1 Analysis codefile R script with information to reproduce analyses presented in this manuscript. (R)
