# Supplementary material for: Authors and editors assort on gender and geography in high-rank ecological publications
Source: PLoS One. 2018 Feb 8;13(2):e0192481. doi: 10.1371/journal.pone.0192481 (PMC5805316; doi:10.1371/journal.pone.0192481)
Supplement: S1 Supporting text — (DOCX) [file pone.0192481.s001.docx]

**Authors and editors assort on gender and geography in high-rank ecological publications**

**SUPPLEMENTARY INFORMATION**

Kezia R. Manlove

Rebecca M. Belou

**1. ADDITIONAL DESCRIPTIONS OF GENDER CLASSIFICATION**

We started with 2,532 papers, of which 2,430 included full lead author first names (the remaining 98 printed only first initials, and were eliminated from the study). The gender classifier successfully categorized 70% of lead author first names (1,696 / 2,430) and 84% of handling editor first names (2,056 / 2,447). Follow-up searches let us classify another 15% of lead authors (accounting for an additional 357 papers), and 11% of handling editors (accounting for an additional 257 papers). 81% of editorial board member names (733 / 904) were classified by the automated gender classifier (distributions of confidence in name classifications are shown in Figure S1). Another 14% (127 / 904) were classified through additional searches, so that 95% of editorial board members were eventually classified to a gender.

232 of the lead authors whose names were classified by our classifiers received an “either” gender classification. This was also true of 193 of the classified handling editor names, and 69 of the editorial board members.

**2. ADDITIONAL DESCRIPTIONS OF GEOGRAPHIC STRUCTURE**

We could determine institutional locations for 2349 of the 2532 lead authors, 2401 of the 2532 handling editors, and 898 of the 904 editorial board members based on their first listed affiliations in publication metadata or on journal websites. Table S1 shows the number of lead authors, editorial board members, and handling editors by continent. A map of author and handling editor contributions by country is shown in Figure S4.

**Table A.** Tabulation of participant continents, as derived from first listed institutional affiliation.

| **Role** | **Africa** | **Asia** | **Australia** | **Europe** | **North America** | **Oceania** | **South America** |
| --- | --- | --- | --- | --- | --- | --- | --- |
| Lead | 17 | 201 | 130 | 904 | 985 | 30 | 57 |
| Board | 1 | 40 | 41 | 259 | 489 | 21 | 11 |
| Handling | 0 | 94 | 117 | 808 | 1252 | 54 | 61 |

**3. ADDITIONAL DESCRIPTIONS OF STATISTICAL MODEL FITS**

Participant gender as a function of role (lead author, editorial board member, handling editor)

We tested the hypothesis that participant gender varied depending on role in the publication process (here, lead author vs. editor) using a logistic regression model with a binary response taking on the value 1 if the participant was female, and 0 if the participant was male. Those responses were modeled as a function of the participant’s role in the publication process, *X_AuthorEditorBoard_* , which was a categorical variable with levels “author”, “board member”, and “handling editor”. Individuals who contributed multiple papers, or who participated in multiple roles were allowed to enter the analysis multiple times. The model was constructed to allow for journal-to-journal variation in gender composition through a hierarchical intercept adjustment. The model is written as follows, and model output is summarized in Table S2:

$$logit\left( Pr\left[ {Participant}_{i}is female \right] \right)={\beta_{0}+\beta}_{1}X_{AuthorEditorBoard}+\beta_{j\left[ i \right]}+\varepsilon_{i}$$

$$\beta_{j}\sim Normal\left( 0,\sigma_{Journal} \right)$$

**Table B.** Model coefficient estimates for a logistic regression testing whether female participation rates varied depending on role (author/board member/handling editor) in the publication process. Journal-to-journal variance (σ_Journal_^2^) was estimated at 0.026.

|  | ***Estimate*** | ***Std. Error*** | ***Z-value*** | ***Pr(>\|z\|)*** |
| --- | --- | --- | --- | --- |
| Intercept (β_0_) | -0.996 | 0.068 | -14.47 | <0.001 |
| Change from author to board member (β_1.1_) | -0.193 | 0.086 | -2.241 | 0.025 |
| Change from author to handling editor (β_1.2_) | -0.499 | 0.067 | -7.481 | <0.001 |

Institutional rank as a function of participant role

We fit a quasi-Poisson model to estimate differences in institutional rank between lead authors, editorial board members, and handling editors, after also adjusting for journal-to-journal variation. Note that this fit assumes a median rank value for institutions ranked from 51-75, 76-100, 101-150, and 151-200 under the Shanghai ranking system for 2016 in the Life Sciences. All unranked institutions were assigned a rank of 250. All ranks were then subtracted from 250, so that the institution with Shanghai ranking = 1 had a response variable value of 249, and all unranked institutions had response variable values of 0. The model statement is as follows, and model output is included in Table S3.

$$\log\left( I{nstitutional Rank}_{i} \right)={\beta_{0}+\beta}_{1}I_{Participanti^{'}srole}+\gamma_{j[i]}+\varepsilon_{i}$$

**Table C.** Model coefficient estimates from a quasi-Poisson model quantifying the relationship between participant role and expected institutional rank, after accounting for journal-to-journal variation (σ_Journal_^2^ = 133.149). The model additionally accounted for overdispersion by using a quasiPoisson family implemented through the glmmPQL function in R’s MASS package.

|  | ***Value*** | ***Std. error*** | ***DF*** | ***t-value*** | ***p-value*** |
| --- | --- | --- | --- | --- | --- |
| ***Expected institutional rank for board members*** | 169.805 | 4.164 | 5636 | 40.7722 | <0.0001 |
| ***Change in expected institutional rank associated with switching role to board member from lead author*** | -1.940 | 3.700 | 5636 | -0.524 | 0.600 |
| ***Change in expected institutional rank associated with switching role to handling from lead author*** | -8.168 | 2.711 | 5636 | -3.013 | 0.003 |

Affiliation in non-G8 country as a function of participant role

We fit a logistic regression model to estimate differences in probability of coming from a non-G8 country between lead authors, editorial board members, and handling editors, after also adjusting for journal-to-journal variation. The model statement is as follows, and model output is included in Table S4.

$$logit\left( Pr\left[ {Participant}_{i} from Non-G8 \right] \right)={\beta_{0}+\beta}_{1}X_{AuthorEditorBoard}+\beta_{j\left[ i \right]}+\varepsilon_{i}$$

**Table D.** Model coefficient estimates for a logistic regression testing whether probability of affiliation with an institution outside of the G8 varied depending on role (author/board member/handling editor) in the publication process. Journal-to-journal variance (σ_Journal_^2^) was estimated at 0.084.

$$\beta_{j}\sim Normal\left( 0,\sigma_{Journal} \right)$$

|  | ***Estimate*** | ***Std. Error*** | ***Z-value*** | ***Pr(>\|z\|)*** |
| --- | --- | --- | --- | --- |
| Intercept (β_0_) | -1.023 | 0.101 | -10.084 | <0.001 |
| Change from author to board member (β_1.1_) | -0.322 | 0.084 | -3.808 | <0.001 |
| Change from author to handling editor (β_1.2_) | -0.485 | 0.061 | -7.926 | <0.001 |

Lead author gender as a function of institutional rank

We used a logistic regression model with hierarchical terms for country-to-country and journal-to-journal variation to estimate the relationships between institutional rank (here, incorporated through an indicator variable showing whether or not an institution was ranked among the top 100) and lead-author gender. The model statement is as follows, and model output is included in Table S5:

$$logit\left( Pr\left[ {Lead author}_{i} is female \right] \right)={\beta_{0}+\beta}_{1}I_{Top100}+\gamma_{j[i]}+\delta_{k[i]}{+\varepsilon}_{i}$$

$$\gamma_{j}\sim Normal\left( 0,\sigma_{Journal} \right)$$

$$\delta_{k}\sim Normal\left( 0,\sigma_{Country} \right)$$

**Table E.** Model coefficient estimates for a logistic regression testing whether the probability that a lead author was female varied depending on whether the author’s institution was ranked among the top 100, after accounting for country-to-country and journal-to-journal variation in probabilities of female participation. The journal-to-journal variance (σ_Journal_^2^) was estimated at 0.073, and country-to-country variance (σ^2^_Country_) was estimated at 0.075. The model was fit on 1897 papers with classified lead-author genders (“either” classifications omitted), including data from 47 countries and 10 journals.

|  | **Estimate** | **Std. Error** | **z value** | **Pr(>\|z\|)** |
| --- | --- | --- | --- | --- |
| **(Intercept)** | 0.527 | 0.124 | 4.251 | <0.001 |
| **Lead author from a top-100 ranked institution** | -0.222 | 0.114 | -1.954 | 0.051 |

Handling editor gender as a function of institutional rank

We fit a logistic regression model describing how the probability a handling editor was female varied with institutional rank. The model had hierarchical terms accounting for country-to-country and journal-to-journal variation. A complete model statement is as follows, and model output is included in Table S6.

$$logit\left( Pr\left[ {Editor}_{i} is from top-100 \right] \right)={\beta_{0}+\beta}_{1}I_{Top100}+\gamma_{j[i]}+\delta_{k\left[ i \right]}+\varepsilon_{i}$$

$$\gamma_{j}\sim Normal\left( 0,\sigma_{Journal} \right)$$

$$\delta_{k}\sim Normal\left( 0,\sigma_{Country} \right)$$

**Table F.** Model coefficient estimates for a logistic regression testing whether the probability that a handling editor was female varied depending on whether the editor’s institution was ranked among the top 100, after accounting for country-to-country and journal-to-journal variation in probabilities of female participation. The journal-to-journal variance (σ_Journal_^2^) was estimated at 0.119, and country-to-country variance (σ^2^_Country_) was estimated at 2.041. The model was fit on 2128 papers with classified handling editor genders (“either” classifications omitted), including data from 32 countries and 10 journals.

|  | ***Estimate*** | ***Std. Error*** | ***z value*** | ***Pr(>\|z\|)*** |
| --- | --- | --- | --- | --- |
| ***(Intercept)*** | **1.314** | **0.317** | **4.144** | **<0.001** |
| ***Editor from a top-100 ranked institution*** | **-0.452** | **0.126** | **-3.604** | **<0.001** |

**4. SUPPLEMENTARY FIGURES**


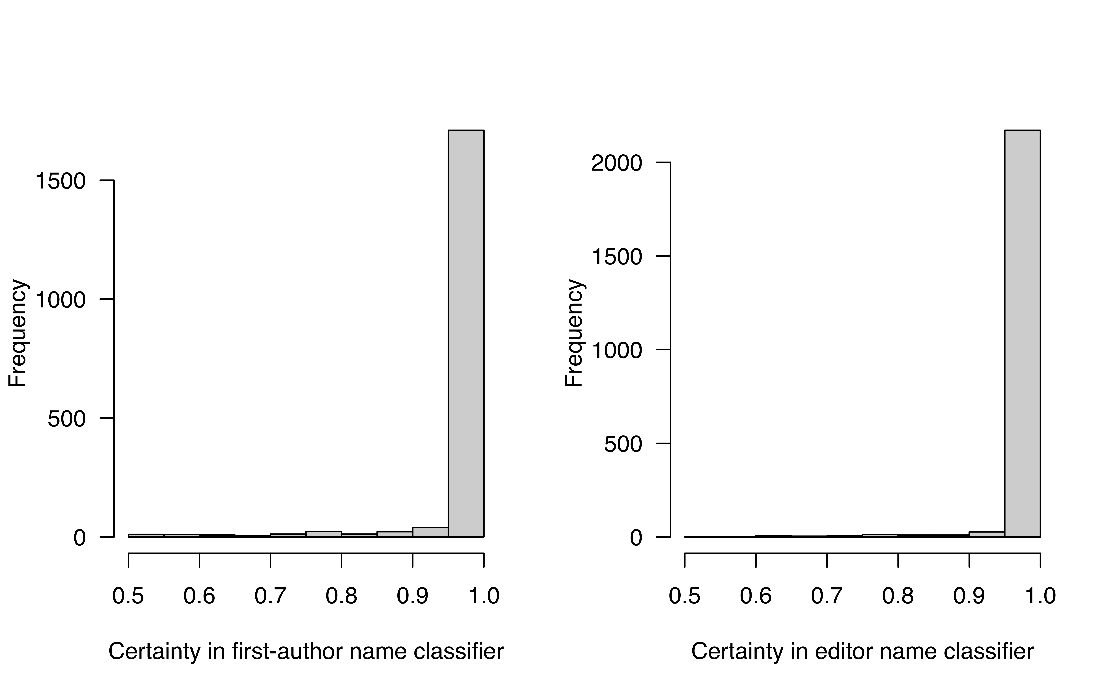


**Figure A.** Distribution of certainties attributed to gender classifications for names classified in our study (using the “ssa” method implemented in R’s gender package). We note that our analyses rely on the kantrowitz method, not ssa, but kantrowitz does not return a probability of correct classification, so we used sea here to attempted to characterize uncertainty. ssa and kantrowitz methods nearly always agreed in classification.


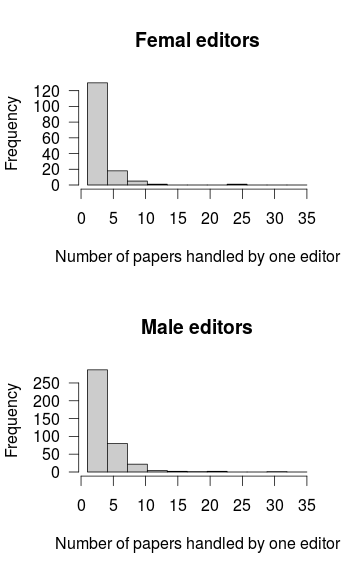


**Figure B.** Distribution of number of papers edited per editor, by editor gender.


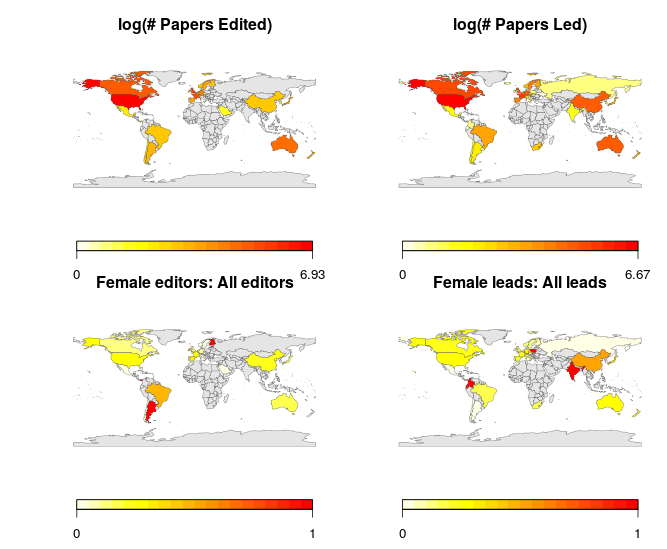


**Figure C.** Geographic distributions of editor and lead author institutions. a) Distribution of papers edited by authors with main affiliations in each country. b) Same as a), but applied to papers led by authors from a particular country. c) Ratio of papers edited by women from this country to all papers edited by editors from this country. d) Same as c), but applied to lead-authored papers. Grey countries contributed no papers to the given classification scheme. Bottom row color ramp is the ratio of female editors (lead authors) to all editors (lead authors). Redder = higher proportion female.


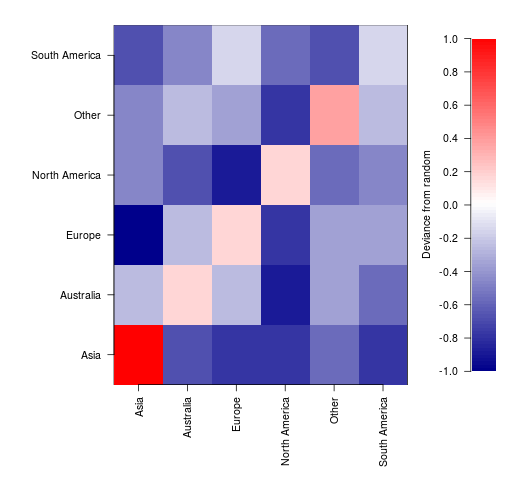


**Figure D.** Assortativity of lead authors and editors by institutional continent. Redder cells are over-represented combinations; bluer cells are under-represented relative to overall expectations based on the marginal distributions of lead-author and editor institutional continent.


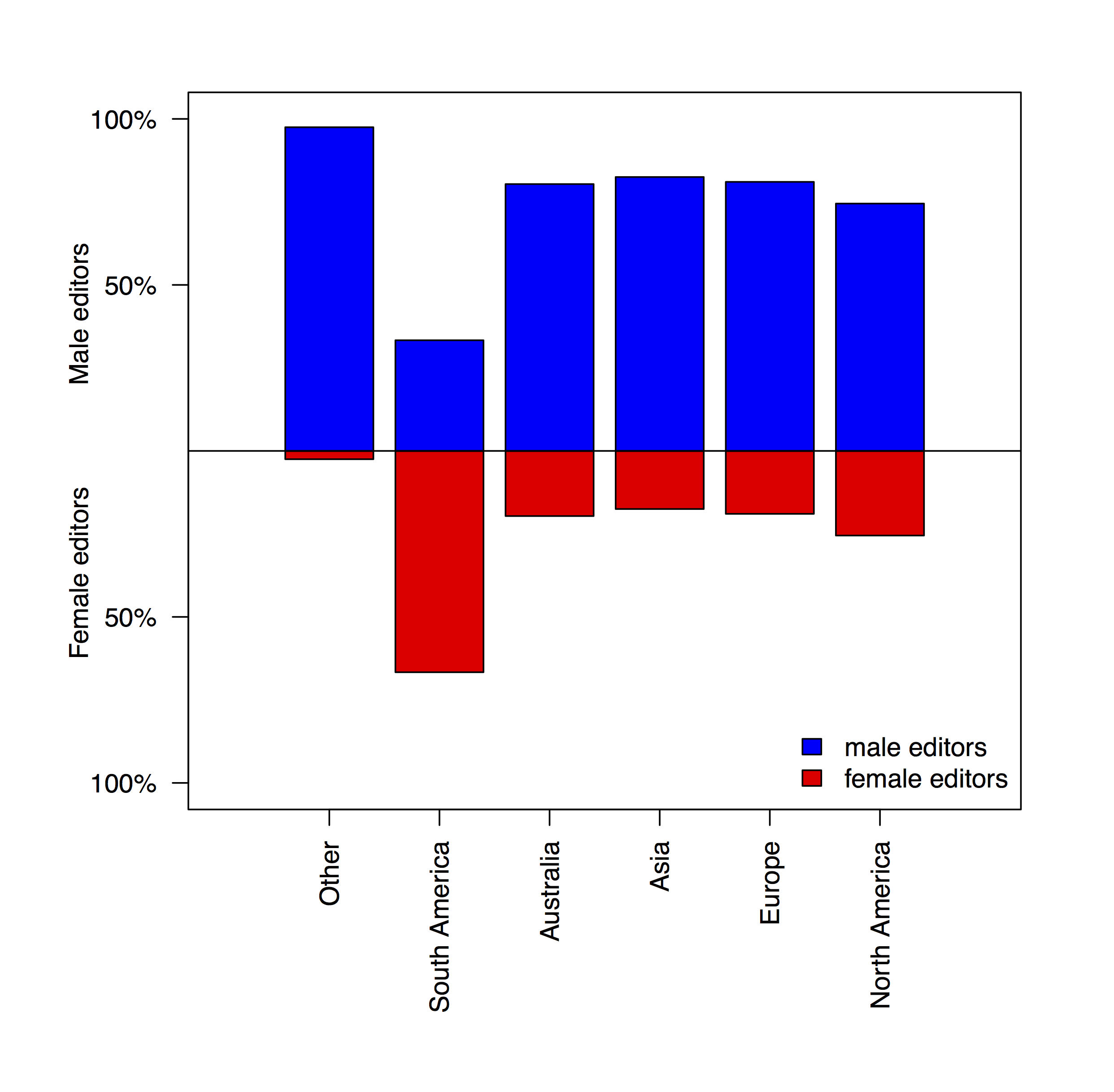


**Figure E.** Percent of handling editors who were male (above the solid line) and female (below the solid line), by continent. Female editors from South America were significantly over-represented.
