# Supplementary material for: Authors and editors assort on gender and geography in high-rank ecological publications
Source: PLoS One. 2018 Feb 8;13(2):e0192481. doi: 10.1371/journal.pone.0192481 (PMC5805316; doi:10.1371/journal.pone.0192481)
Supplement: S1 Data file — (DOCX) [file pone.0192481.s002.docx]

Ecological metadata file for datasets used in Manlove, K. R. & Belou, R. (2018). “Authors and editors assort on gender and geography in high-rank ecological publications.” *PLoS One.*

| **General metadata** |  |
| --- | --- |
| Abstract | These data were collected to investigate whether editor features (gender, institutional affiliation’s rank or geographic location) assort non-randomly with lead author features for publications in high-impact ecological journals |
| Keywords | Ecological publishing, peer review, women in STEM, minorities in STEM, institutional rank |
| Usage rights | This dataset is published as supplementary information in the manuscript “Authors and editors assort on gender and geography in high-rank ecological publications” (full citation information included below). |
| Funding | This research was conducted without support from any grant or institution. |
| Individual: Owner | Kezia R Manlove |
| Position | Postdoctoral researcher |
| Address | 2917 Oliver St. Bozeman, MT, 59718 |
| Phone | (406) 600-2369 |
|  |  |
| Email | [kezia.manlove@gmail.com](mailto:kezia.manlove@gmail.com) |
| Web address | [www.keziamanlove.com](http://www.keziamanlove.com) |
|  |  |
| Individual: Primary contact | Kezia R Manlove |
| Position | Postdoctoral researcher |
| Address | 2917 Oliver St. Bozeman, MT, 59718 |
| Phone | (406) 600-2369 |
|  |  |
| Email | [kezia.manlove@gmail.com](mailto:kezia.manlove@gmail.com) |
| Web address | [www.keziamanlove.com](http://www.keziamanlove.com) |
| Organization | Washington State University |
|  |  |
| **Article citation** |  |
| Author | Kezia R Manlove & Rebecca Belou |
| Date |  |
| Title | Authors and editors assort on gender and geography in high-rank ecological publications |
| Journal | PLoS One |
| Volume |  |
| Issue |  |
| Page range |  |
| **Geographic metadata** |  |
| Geographic description | Geographic information for editor and author institutions were extracted from wikipedia, using the GeoHack functionality. We recorded coordinates in decimal degrees, and did not rely on a particular projection. |
| Bounding coordinates | N/A — coordinates spanned the globe |
| **Temporal metadata** |  |
| Temporal description | Articles in this study were published between January of 2015 and November of 2016. Most journals in the study published on a monthly basis, so articles arose systematically within that timeframe. |
| Begin | January, 2015 |
| End | November, 2016 |
| **Taxonomic metadata** |  |
| Taxonomic authority | N/A |
| **Methods metadata** |  |
| General sampling design | We censused the published literature in the ten study journals using Web of Science. For journals with more than 300 papers published in our timeframe, we systematically sampled the papers through time to reach a sample at or near 300 papers. |
| Participant genders | We used a gender classifier in R's gender package to classify most of the participants to a particular gender (most classifications occurred with high levels of confidence — see published text for details). When the gender classified failed to successfully classify a participant, we used a variety of baby naming sites. While these sites have some caveats, they allowed us to classify many non-western names. We felt that the bias associated with excluding non-western names was greater than the bias possibly introduced through use of these various sites. |
| Institutional Affiliation | We used institutional affiliations associated with each participant (either institutional affiliation information on the publication itself for the authors, or as documented on editorial board pages for editors). When individuals listed multiple affiliations, we relied only on the first affiliation listed. |
| Quality control | Paper were extracted from Web of Science by KRM during the week of November 15, 2016. Editorial board data were extracted from journal editorial board websites between November 20th and December 2st, 2016. We tabulated the data multiple ways to cross-check for inconsistencies. All gender classifications were checked by hand, as were institutional rank values. |
| **Data table metadata: FinalPaperData_20171217.csv** | |
| File name | FinalPaperData_20171217.csv |
| Case sensitive? | Yes |
| Number of records | 2533 |
| Orientation | Observations (here, sampled papers) are in rows. |
|  |  |
| **Data table metadata: Institutions_20170102.csv** | |
| File name | Institutions_20170102.csv |
| Case sensitive? | Yes |
| Number of records | 1085 |
| Orientation | Observations (here, all institutional affiliations of authors, editors, or editorial board members) are in rows. |
|  |  |
| **Data table metadata: BoardMembers_20170201.csv** | |
| File name | BoardMembers_20170201.csv |
| Case sensitive? | Yes |
| Number of records | 904 |
| Orientation | Observations (here, editorial board members extracted from journal editorial board web pages) are in rows. |
|  |  |

| Data table structure and attribute description: FinalPaperData_20171217.csv | | | | | |
| --- | --- | --- | --- | --- | --- |
| Attribute name | Description | Unit | Type | Missing | Precision |
| Title | Paper's title |  | String |  |  |
| Source | Name of journal where paper was published |  | Factor (levels are names of included journals) |  |  |
| LeadAuthorFirst | Lead author's first name, as reported on the published paper. |  | String |  |  |
| LeadAuthorLast | Lead author's last name, as reported on the published paper. |  | String |  |  |
| LeadInitOnly | yes/no variable; equals yes when lead authors' initials were published, but not their full names |  | Factor (yes/no) |  |  |
| first.gender | Initial lead author gender classification based on the gender package in R |  | Factor (male/female) | NA |  |
| first.gender.manual | Manually updated gender classifications that included names classified based on other sources |  | Factor (male/female) | NA |  |
| first.gender.manual.method | Sources for manually classified first names |  | String | (NA or blank) |  |
| EditorFirst | Editor's first name, as published on the manuscript |  | String | (NA or blank) |  |
| EditorLast | Editor's last name, as published on the manuscript |  | String | (NA or blank) |  |
| editor.gender.manual | Manually revised editor gender classifications. |  | Factor (male/female) | (blank) |  |
| FirstInst2 | Lead author's institutional affiliation |  | Factor (Institution names -- these were standardized across languages and different versions of the same name, e.g., Penn State was recoded to Pennsylvania State University) | (blank) |  |
| Editor.Institution | Editor’s institutional affiliation |  | Factor (institutional affiliations) | (blank) |  |

| Data table structure and attribute description: Institutions_20170102.csv | | | | | |
| --- | --- | --- | --- | --- | --- |
| Attribute name | Description | Unit | Type | Missing | Precision |
| Editor Institutions | Institutional affiliation's name. Institutional names were edited in the FinalPapers csv when the same institution was referenced under multiple names. |  | String | (blank) |  |
| ShanghaiRank2016 | Institution’s ranking according to the Shanghai index in 2016 |  | String (recoded to numeric in the script files) | (blank) |  |
| Editor City | Institution’s city |  | Factor (levels = cities with institutions) | (blank) |  |
| Editor State | Institution's state |  | Factor (levels = states with institutions) | (blank) |  |
| Editor Country | Institution's country |  | Factor (levels = countries with institutions) | (blank) |  |
| WGS84Lat | Latitude (in decimal degrees, extracted from Wiki’s geoHack functionality) | decimal degrees |  | (blank) | Provided by geoHack; varies by institution |
| WGS84Long | Longitude (in decimal degrees, extracted from Wiki’s geoHack functionality) | decimal degrees |  | (blank) | Provided by geoHack; varies by institution |

| Data table structure and attribute description: BoardMembers_20170201.csv | | | | | |
| --- | --- | --- | --- | --- | --- |
| Attribute name | Description | Unit | Type | Missing | Precision |
| Journal | Name of journal with which editor is affiliated |  | Factor (levels are studied journals) |  |  |
| First | Editor's first name |  | String |  |  |
| Last | Editor's last name |  | String |  |  |
| Institution | Editor's institutional affiliation (sometimes edited from what is listed on the editorial board page to comply to institutional names in Institutions_20170102.csv). |  | Factor (levels are Institution names in Institutions_20170102.csv). | NA |  |
| gender | Editor's gender classification using R’s gender package |  | Factor (male/female) | NA |  |
| gender.manual | Manually revised editor gender classification |  | Factor (male/female) | NA |  |
| gender.manual.source | Source for manual revisions to gender. |  | String | (blank) |  |
